# Global choroidal thickness in healthy eyes assessed by ultra-widefield swept-source optical coherence tomography

**DOI:** 10.1007/s00417-026-07234-z

**Published:** 2026-04-15

**Authors:** Ceren Soylu, Alberto Quarta, Jianfeng Huang, Giulia Corradetti, Mai Alhelaly, Rouzbeh Abbasgholizadeh, Shinichiro Chujo, Hyun Duck Kwak, Yu-Chien Chung, Giacomo Boscia, Raiyna Rattu, Muneeswar G. Nittala, Jane W. Chan, SriniVas R. Sadda

**Affiliations:** 1 Doheny Eye Institute, Pasadena, CA USA; 2 Department of Ophthalmology, David Geffen School of Medicine, University of California, Los Angeles, CA USA; 3 Beijing Hospital, National Center of Gerontology, Institute of Geriatric Medicine, Chinese Academy of Medical Sciences, Beijing, 100730 P.R. China; 4 Ophthalmology Department, Tanta University, Tanta, Egypt; 5 Department of Ophthalmology, Mie University Graduate School of Medicine, Tsu City, Mie Japan; 6 Department of Ophthalmology, Fu Chen Catholic University Hospital, Fu Jen Catholic University, New Taipei, New Taipei City, Taiwan; 7 Department of Translational Biomedicine Neuroscience, University of Bari “Aldo Moro”, Bari, Italy; 8 Doheny Image Reading and Research Laboratory, Doheny Eye Institute, 150 N. Orange Grove Blvd, Suite 232, Pasadena, CA 91103 USA

**Keywords:** Choroidal thickness (CT), Ultra-widefield (UWF), Swept-source optical coherence tomography (SS-OCT), Optic nerve head (ONH), Axial length (AL)

## Abstract

**Purpose:**

To evaluate differences in choroidal thickness (CT) throughout the fundus using ultra-widefield swept-source optical coherence tomography (UWF SS-OCT) and to assess its association with age, sex and axial length (AL).

**Methods:**

Seventy-two eyes of 36 healthy subjects were imaged with an UWF SS-OCT using a 26 mm x 21 mm volume scan protocol. Mean CT by quadrant was computed within concentric rings centered on the optic nerve head (ONH). CT values were then plotted in 4 quadrants as a function of distance from ONH. Mean CT of each quadrant was correlated with age, sex and AL.

**Results:**

All 4 quadrants demonstrated a rapid initial rise in CT with the superior, inferior, and nasal quadrants reaching peak values at 3.75 mm from the ONH center. The temporal quadrant peaked at 5.25 mm with the highest CT overall (341.41 ± 69 µm). The inferior quadrant had the lowest CT at 8.25 mm (177.62 ± 36.5 µm). In the temporal quadrant, CT was negatively associated with age and AL (both *p* < 0.05) and was thinner in females (*p* = 0.036). Superior CT was negatively associated with AL (*p* = 0.007). Inferior CT was associated with age and AL (both *p* < 0.05), with no impact of sex.

**Conclusion:**

CT exhibited regional variation throughout the retina with the temporal quadrant demonstrating the greatest thickness and the inferior quadrant showing the lowest CT. Axial length was the most consistent determinant of CT across multiple quadrants while age and sex demonstrated quadrant-specific effects.

## Key Messages

*****What is known***:**

- Ultra-widefield swept-source optical coherence tomography (SS-UWF OCT) allows the imaging of the peripheral choroid, however normative data describing global choroidal thickness (CT) distribution and its anatomic determinants remain limited.
- CT varies regionally throughout the retina but most prior studies relied on fovea-centered measurements.

*****What is new***:**

- This study introduces optic nerve head (ONH) as a more anatomically accurate reference point in CT mapping rather than fovea-centered measurements, due to the closure of the optic cup inferior to optic nerve.
- Axial length (AL) emerges as the most consistent determinant of CT across the retina, while age and sex exhibit quadrant-dependent effects.

## Introduction

The choroid is the posterior vascular component of the uvea, located between the retina and sclera. It serves multiple functions, including supplying oxygen and nutrients to the outer retina, modulating retinal position through dynamic changes in choroidal thickness (CT), regulating ocular temperature, and secreting various growth factors [1]. Choroidal and choriocapillaris abnormalities have been implicated in various chorioretinal diseases such as age-related macular degeneration, central serous chorioretinopathy, Vogt-Koyanagi-Harada disease and pathologic myopia [2–8].

Although visualization of the choroid was previously challenging due to signal scattering, the development of enhanced depth imaging optical coherence tomography (OCT) enabled improved delineation of the choroidal architecture [9]. Swept-source (SS) OCT further advanced choroidal imaging by providing faster acquisition speed, wider scan area and deeper tissue penetration [10]. Most previous studies focused on CT in the macular region in various conditions [11–14]. However, the advent of ultra-widefield (UWF) SS OCT, providing fields of view up to 180°–200° has enabled visualization of the peripheral choroid and yielded new insights into its topographic characteristics [15–20].

The scarcity of normative data on peripheral CT poses challenges for interpreting peripheral choroidal changes in chorioretinal diseases. Moreover, discrepancies in CT measurements among healthy eyes due to differences in imaging devices and measurement methodologies further complicate comparisons across studies. Establishing a standardized normative CT database and computing a mathematical model for CT through sectors would be essential for accurate assessment of choroidal changes in chorioretinal diseases.

In this study, we used UWF SS-OCT device (VG200D, Intalight Inc., San Jose, CA, USA) to evaluate the change in CT as a function of distance from optic nerve head (ONH) throughout the fundus and to assess its association with age, sex and axial length.

## Methods

This prospective, cross-sectional study was conducted at the Doheny-UCLA Eye Center in Pasadena. Written informed consent was obtained from all participants. The study received approval from the Institutional Review Board of University of California Los Angeles and was conducted in accordance with the Declaration of Helsinki.

### Study participants

Seventy-two normal eyes of 36 healthy adults were imaged between March and May 2025. Best corrected visual acuity (BCVA), refractive error and axial length (AL) (IOL Master 500, Zeiss Meditech) were measured. The inclusion criteria were (1) refractive error ≤ -6.00 D, astigmatism < 2 D (2) BCVA (expressed in Snellen visual acuity) ≥ 20/25 (3) image quality score ≥ 7/10 (signal strength index). Exclusion criteria were (1) any systemic (diabetes mellitus, systemic hypertension, cardiovascular disease, autoimmune or inflammatory disorders, neurological diseases) or ocular diseases that could affect choroidal structure (2) history of ocular surgery, and (3) motion artifacts preventing high quality imaging. Both eyes of each patient were included in the study if eligible. Refraction data were converted to spherical equivalent (SE) which was the spherical dioptric power plus half of the cylindrical dioptric power.

### Imaging procedure

All subjects were imaged using a 200-kHz UWF SS-OCT (VG200D, Intalight Inc., San Jose, CA, USA) with a central wavelength of 1050 nm, axial optical resolution of 3.8 μm, lateral optical resolution of 10 μm and scanning depth of 6.3 mm. Scanning protocol was 1536 A-scans x 1240 B-scans which corresponded to a 26 mm horizontal x 21 mm vertical dimensions. Scans were acquired centered on fixation at the foveal center, per the standard practice in the imaging unit.

Data collection was performed by the same trained examiner (CS) for all subjects. The inner and outer choroidal boundaries (defined to be Bruch’s membrane and the choroid–scleral interface, respectively) were segmented using the instrument’s automated segmentation algorithm, and manual correction of segmentation errors was performed as necessary by a single experienced grader (CS) using a standardized grading protocol to ensure consistency across all scans. Choroidal thickness was computed automatically throughout the fundus using concentric rings centered on optic nerve head (ONH).

### Grading protocol

**Fig. 1:**
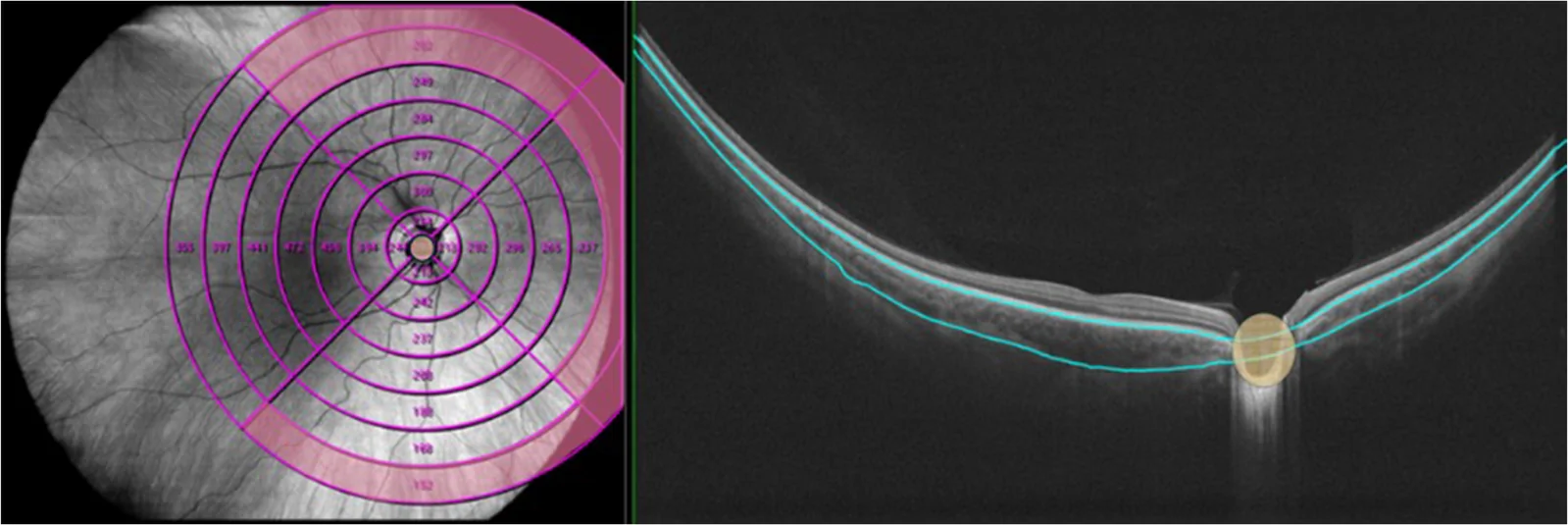
Choroidal thickness analysis strategy. A concentric ring grid is applied on the OCT en face image centered on the optic nerved head. Pink shaded rings which extend beyond the scan’s field of view and are not included in the analysis. The yellow shaded zone, highlights the optic nerve and demonstrates how the segmentation boundaries were extrapolated across the nerve from visible choroid on either side. Such an approach mitigates artifact in choroidal thickness in the inner rings due to variability in the size of the optic nerve

Mean CT by quadrant was computed within concentric rings centered on the ONH. At the ONH, segmentation boundaries were interpolated from the margins of visible choroid. The innermost ring measured 1 mm in diameter, with subsequent rings expanding toward the periphery with diameters of 3,6,9,12,15,18 and 21 mm. Rings which extended beyond the scan’s field of view for a given quadrant were excluded from the analysis for that quadrant. Thus, the two outermost rings in the superior and nasal quadrants and the outermost ring in the inferior quadrant were omitted from all measurements (Fig. 1).

The eccentricity of the rings was computed based on the distance from the ONH (0 mm) center. Choroidal thickness values were then plotted in various quadrants as a function of eccentricity. Mean CT of each quadrant was correlated with age, sex and AL.

### Statistical analysis

All analyses were performed using R version 4.1.0 (R Foundation for Statistical Computing, Vienna, Austria). Continuous variables were assessed for normality using the Shapiro–Wilk test and visual inspection of histograms and Q–Q plots. Normally distributed data are presented as mean ± standard deviation, while non-normal data are summarized using medians and interquartile ranges. Comparisons of CT among retinal quadrants were conducted using repeated-measures analysis of variance (ANOVA). When significant global effects were observed, pairwise comparisons were performed with Bonferroni adjustment to control for multiple testing. Peak CT values across eccentricities were evaluated descriptively and compared using the same repeated-measures framework. Spearman correlation coefficients (ρ) were calculated to assess associations among quadrants. Linear mixed-effects models were used to examine the associations between choroidal thickness and age, sex, and axial length, with a subject-specific random intercept to account for within-participant correlation. Model assumptions were satisfied. Fixed-effect β coefficients are reported with corresponding p-values with statistical significance defined as *p* < 0.05.

## Results

In this study, 72 eyes from 36 healthy subjects were imaged. Of these, 68 eyes from 36 subjects met the inclusion criteria for the analysis. Four eyes were excluded due to poor image quality. The mean age was 40.6 ± 10.3 years, and 18 were males. The mean AL was 24.6 ± 1.1 mm and SE was − 1.7 ± 1.6 D (Table 1).

**Table 1:** Demographics of healthy subjects

Subjects, *n*	36
Eyes, n	68
Gender (female/male), n (%)	18 (50) / 18 (50)
Ethnicity	
Caucasian, n (%)	15 (41.7)
Asian, n (%)	13 (36.1)
Hispanic, n (%)	8 (22.2)
Age, years (mean ± SD)	40.6 ± 10.3
Axial length (mm) (mean ± SD)	24.6 ± 1.1
Spherical equivalent (D) (mean ± SD)	-1.7 ± 1.6

*SD* standard deviation

**Fig. 2:**
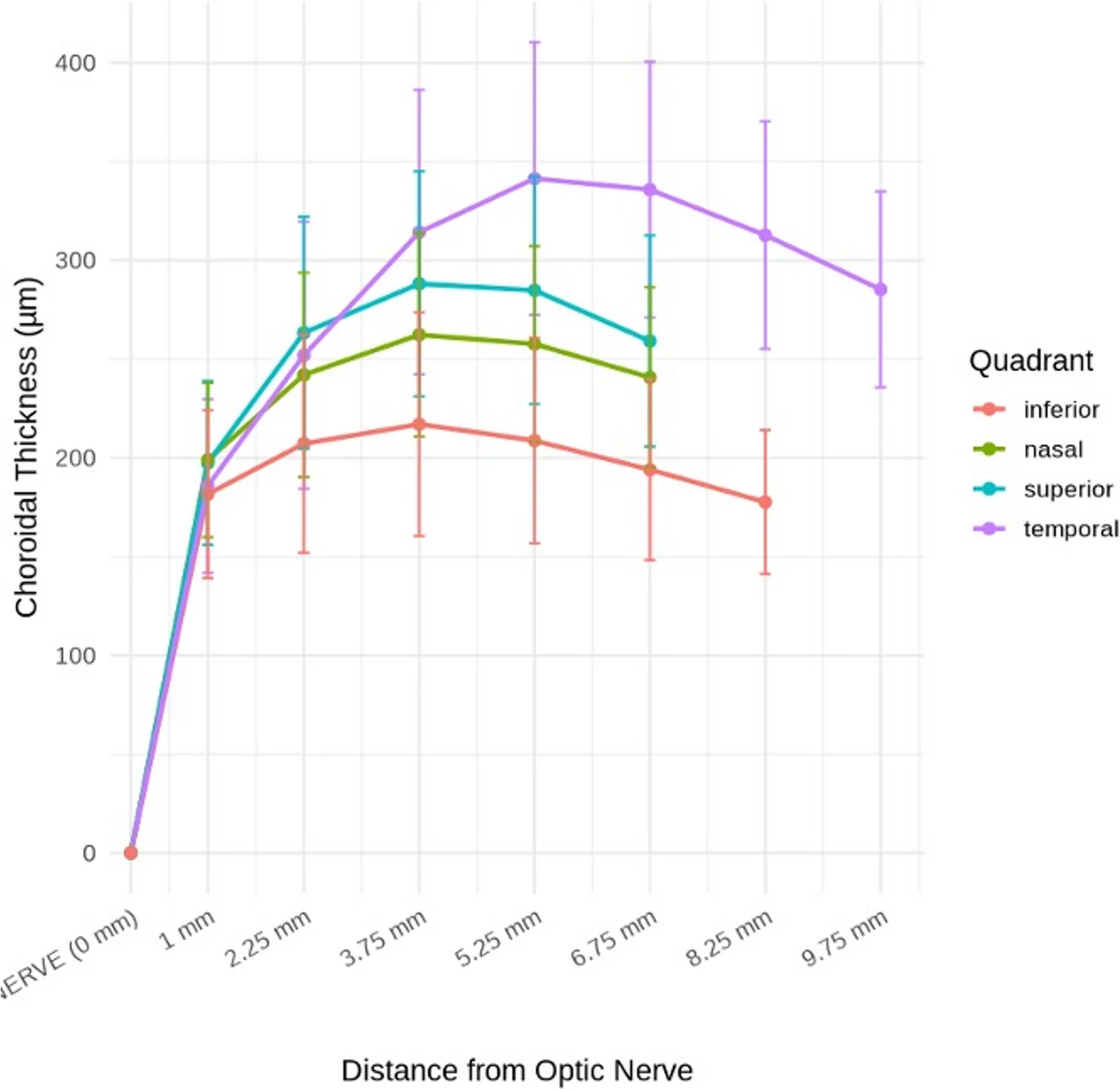
Change in choroidal thickness (CT) as a function of distance from optic nerve head (ONH). Note, the distance from the optic nerve for which data are available vary by quadrant due to the positioning of the scan acquisition over the foveal center. Despite this, the distance at which the CT peak is reached in every quadrant is apparent. While it is reached by 3.75 mm in the inferior, nasal, and superior quadrants; the peak is not reached until 5.25 mm in the temporal quadrant, highlighting the influence of the macula. The overall shape profile to the curves is similar in all quadrants

The mean CT differed significantly among all retinal quadrants, with the greatest thickness observed in the temporal quadrant (289.6 ± 55.0 μm), followed by the superior (258.6 ± 36.5 μm), nasal (240.3 ± 25.0 μm), and inferior (197.7 ± 15.9 μm) quadrants (all pairwise comparisons, *p* < 0.001). All 4 quadrants demonstrated a rapid initial rise in CT with superior, inferior and nasal quadrants reaching their peak CT at 3.75 mm (288.12 ± 57, 217.04 ± 56.6 and 262.32 ± 51.5 μm respectively) from the ONH center. The temporal quadrant reached its peak CT at 5.25 mm, also demonstrating the highest CT (341.41 ± 69 μm) among the four. The inferior quadrant had the lowest CT value 177.62 ± 36.5 μm which was reached at a distance of 8.25 mm from the ONH (Fig. 2).

**Fig. 3:**
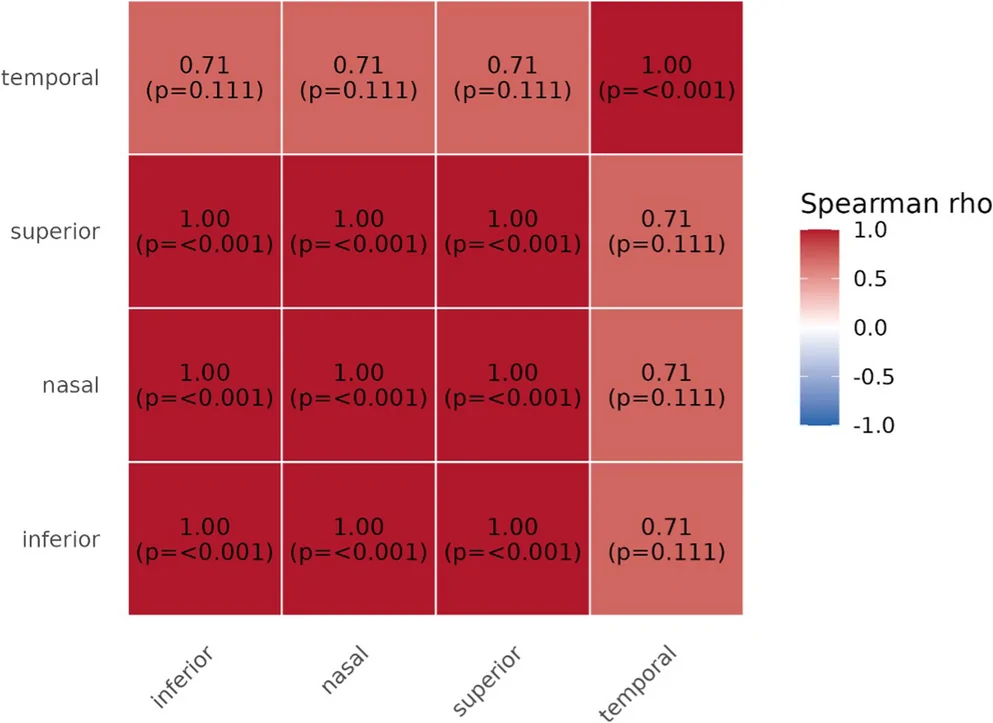
Correlation in choroidal thickness between quadrants. Although there is strong correlation among the superior, nasal, and inferior quadrant CTs when an optic nerve head – centered analytical approach is used, the CT in these quadrants do not correlate with the temporal quadrant. This is presumably due to the influence of the macula in the temporal quadrant

An excellent correlation was observed between superior, inferior and nasal quadrants (ρ = 1.00, *p* < 0.001). The temporal quadrant, however, was not statistically significantly correlated to the other quadrants (ρ = 0.71, *p* = 0.11) (Fig. 3).

In the temporal quadrant, CT showed a significant negative association with age (β = −1.96, p = 0.021) and AL (β = −28.59, p = 0.0001). Female sex was also associated with significantly thinner temporal CT compared with males (β = −35.83, p = 0.036). In the superior quadrant, CT showed a significant negative association with AL (β = −18.21, p = 0.0067). In the inferior quadrant, CT was significantly associated with both age (β = −1.59, p = 0.027) and AL (β = −17.20, p = 0.0011), whereas sex was not a significant predictor (p = 0.206) (Table 2). Since AL and refractive error have are strongly correlated, refraction was excluded from analyses including AL.

**Table 2:** Mean Choroidal Thickness association with age, sex and axial length

Quadrant	Predictor	Estimate	Std. Error	*p*-value
Temporal	Intercept	1128.21	173.73	< 0.001
	Age	–1.96	0.81	0.021
	AL	–28.59	6.34	0.0001
	Sex (F vs. M)	–35.83	16.43	0.036
Superior	Intercept	788.67	170.95	0.0001
	Age	–1.29	0.73	0.087
	AL	–18.21	6.27	0.0067
	Sex (F vs. M)	–19.05	14.85	0.208
Nasal	Intercept	448.92	150.50	0.0053
	Age	–1.33	0.68	0.059
	AL	–5.37	5.50	0.337
	Sex (F vs. M)	–13.97	13.75	0.317
Inferior	Intercept	714.44	131.66	< 0.001
	Age	–1.59	0.69	0.027
	AL	–17.20	4.77	0.0011
	Sex (F vs. M)	–17.86	13.86	0.206

*AL* axial length

## Discussion

In this study evaluating the global choroid using UWF SS-OCT, we demonstrated that CT exhibits regional variation in healthy eyes, with temporal quadrant being the thickest followed by superior and nasal quadrants, and inferior quadrant demonstrating the lowest CT. Though previous studies using UWF SS-OCT have identified a similar regional difference [15, 16, 21, 22], they did not evaluate CT change as a function of distance and they used fovea-centered thickness maps, disregarding the embryological nature of the eye. Optic nerve head serves a more accurate center for CT measurements since the closure of the optic cup ends inferior to optic nerve [23, 24]. The selection of the ONH as the reference point is further validated by the fact the overall shape of the curves is similar across the various quadrants. At the same time, our distance-based analysis also revealed important differences in the choroidal geometry as the peak CT was achieved at different eccentricities, most notably temporally.

Earlier studies conducted before the availability of UWF SS-OCT demonstrated that the choroid was thickest in the macular and supratemporal regions and thinnest inferior to the optic disc; however, these observations were derived from relatively small imaging areas [24–28]. The wider scanning capability of UWF SS-OCT has expanded choroidal assessment to include peripheral regions that were previously inaccessible with conventional imaging. Liu et al. [16] investigated CT variation in 210 eyes using UWF SS- OCT with 24 mm x 20 mm scanning mode and found that thickest mean CT was located at the macular and supratemporal region followed by the nasal side of the optic disc and thinnest below the optic disc, similar to our findings. Hirano et al. [21] also showed in 123 eyes using 23 mm x 20 mm UWF SS-OCT that peripheral CT was significantly lower than the central CT, with nasal being lower than the temporal field and inferior being lower than the superior field.

In our cohort, the temporal quadrant exhibited a distinct spatial pattern of choroidal thickening, reaching its peak thickness at 5.25 mm from the optic nerve head corresponding roughly to the macular region and demonstrating the highest mean CT among all quadrants (341.41 ± 69 μm). In contrast, the superior, nasal, and inferior quadrants reached their maximal thickness at a closer eccentricity of 3.75 mm. Despite its greater absolute CT, the temporal quadrant did not show a statistically significant correlation with the other quadrants (ρ = 0.71, *p* = 0.11), suggesting an independent regional behavior. This distribution is anatomically plausible, as the temporal short posterior ciliary arteries enter the globe in the macular region and spread out toward the peripheral fundus [29]. In parallel, the macula contains the highest density of cone photoreceptors [30] and therefore exhibits the greatest metabolic and oxygen demand, which is supported by a richer choroidal vascular network than in peripheral retinal regions. Overall, we theorize that the different behavior of the CT thickness trajectory is driven by the influence of the macula. However, this observation should be interpreted with caution, as the correlation analysis was based on a limited number of distance points, which may reduce statistical power to detect significant associations.

Consistent with prior reports, the inferior quadrant had the lowest CT [16, 21–23, 28]. This pattern may be explained by the embryologic site of optic fissure closure, with the relative choroidal thinning inferonasal to the optic nerve head representing a remnant of normal ocular development [23, 24]. Due to the reproducible thinning observed in this region, this would again highlight the importance of using the ONH as a more reliable anatomic landmark or reference than the foveal center for assessing regional choroidal thickness variation across the retina [24].

Choroidal thickness has been shown to vary significantly with age, AL, sex, refractive error, and diurnal fluctuations [26, 31, 32]. In our study, temporal quadrant CT was significantly and inversely associated with age (*p* = 0.021), AL (*p* = 0.0001), and female sex (*p* = 0.036). Superior quadrant CT showed a significant negative relationship only with AL (*p* = 0.0067), whereas inferior quadrant CT was inversely associated with both age (*p* = 0.027) and AL (*p* = 0.0011). The literature reports variable results regarding these associations. Hirano et al. [21] demonstrated a significant inverse correlation between age and CT across all subfields whereas AL showed a significant negative association only in the central and inferonasal subfields. Notably, the effect of age on CT was stronger than that of AL. Liu et al. suggested that after age 50, mean CT was significantly and negatively correlated with age [16]. Ouyang et al. [24] demonstrated a significant inverse association between AL and CT both subfoveally and across all Early Treatment Diabetic Retinopathy Study (ETDRS) subfields. Age was likewise a significant determinant of CT subfoveally and in most macular subfields, but not in the temporal inner and outer rings. Ikuno et al. [26] identified age and myopic refractive error as independent factors significantly associated with choroidal thinning. The association between sex and CT has been variably reported in the literature. Several studies have demonstrated thicker choroid in males, whereas others have observed either no significant differences or region-specific variations between sexes [33–35]. Barteselli et al. reported significantly greater macular choroidal volume in men compared with women [33] and Mori et al. similarly demonstrated thicker choroid in men younger than 50 years [35]. Hormonal influences, particularly the vascular and metabolic effects of estrogen and androgens, have been proposed as potential contributors to sex-related differences in choroidal structure and perfusion [17, 36]. Estrogen-mediated vascular regulation and age-related hormonal decline may influence choroidal blood flow and thickness in a regionally variable manner. In this context, the observed thinning in the temporal quadrant among females in our cohort may reflect localized vascular or hormonal influences on the temporal choroid. In our study, AL was the most consistent determinant of CT across multiple quadrants. The observed variability among studies may be attributable to heterogeneity in cohort characteristics as well as differences in CT acquisition and analysis protocols.

Assessing CT throughout the retina offers a more comprehensive and practical evaluation of choroidal architecture than measurements in the macular region alone. Such regional profiling has direct clinical implications, since localized patterns of choroidal changes may help in disease phenotyping, improve monitoring of progression, and refine assessment of treatment response in chorioretinal disorders. Moreover, the use of an anatomically meaningful reference allows for more precise and reproducible mapping of CT distribution. Collectively, these attributes underscore the strong clinical applicability of our approach. Going forward, we would advocate for an ONH-centered analytical strategy considering distance from the ONH and the clock-hour meridian as key attributes when describing choroidal thickness. Using such an approach may allow greater harmonization of results across studies.

Our study has a number of limitations. First, we had a relatively small cohort and narrow age distribution which may limit the generalizability of our results. Although the cohort size is comparable to prior UWF OCT studies of CT, our cohort does not fully capture the broader demographic spectrum, particularly older individuals who are more likely to develop chorioretinal disease. Accordingly, this study should be considered a pilot normative characterization of global choroidal thickness using an ONH–centered framework. Second, other factors that could potentially influence CT, such as diurnal variation and systemic blood pressure, were not controlled for. This temporal variability may introduce additional dispersion when establishing normative values. As this study represents a pilot normative characterization, such variability should be considered when interpreting the absolute measurements. Third, manual correction of automated choroidal segmentation was performed by a single experienced grader to ensure consistency across all scans; however, formal inter- and intra-grader reproducibility metrics were not assessed. The absence of reproducibility analysis represents a limitation, particularly for a study proposing normative reference values. Future studies on normative datasets may incorporate agreement analyses to validate measurement reliability. Fourth, we interpolated segmentation boundaries from the margins of visible choroid at ONH which created a thickness value at the optic nerve center, though there is no choroid present there. An alternate approach would have been to exclude or mask this area. However, such an approach would have introduced a variability in the thickness measurements of the inner most rings depending on the size of the optic nerve among subjects. The innermost ring represented only a small portion of the analytical area and did not influence identification of peak choroidal thickness or the primary spatial analyses. Future studies may consider alternative approaches, such as masking the ONH region, adjusting for disc size, or performing sensitivity analyses, to further evaluate the impact of this methodology. Fifth, because the scans were acquired centered on the fovea, but the analysis was centered on the optic nerve, some of the outermost rings could not be analyzed for some quadrants as they fell beyond the field of view of OCT scan acquisition. Future studies aimed at evaluating the choroid may consider centering the acquisition over the optic nerve head. Despite these limitations, our study precisely characterized regional variations in CT as a function of distance using a more anatomically appropriate reference point.

In summary, our findings suggest that CT displays regional heterogeneity across the retina, peaking in the temporal quadrant and reaching its lowest values in the inferior quadrant. Axial length is the most uniform predictor of CT throughout the retina, whereas age and sex depend on the quadrant. Further studies with larger and more diverse cohorts are warranted to validate these findings. The development of a standardized normative reference for CT in UWF SS-OCT and a consistent reference-based analytical strategy is essential for the precise assessment of choroidal changes in chorioretinal diseases.

## Data Availability

No datasets were generated or analysed during the current study.

## Abbreviations

AL: axial length
BCVA: best corrected visual acuity
CT: choroidal thickness
SS: swept-source
OCT: optical coherence tomography
ONH: optic nerve head
SE: spherical equivalent
SS: swept-source
UWF: ultra-widefield
